# Medicine, Pathological, Practical, and Therapeutical

**Published:** 1836-07

**Authors:** 


### MEDICINE,

### PATHOLOGICAL, PRACTICAL, AND THERAPEUTICAL.

### *On Thymic Asthma.* By Dr. HIRSCH, of Kœnigsberg.

[DR. KOPP read a paper on this subject at the annual meeting of scientific men at Heidelberg, which has since been published in a separate work by the author, (*Denkwurdigkeiten in der ärtzlichen Praxis.* Bd. i. *Frankfurt,* 1830.) In the present memoir Dr. Hirsch has collected some additional cases, and has drawn up a digested account of the whole disease from collation of the various memoirs published on the subject in Germany, since Dr. Kopp first called particular attention to it. We shall give, in the first place, an abridgment of Dr. Kopp's original cases, and then an abstract of Dr. Hirsch's general observations.]

CASE 1. A delicate woman, with weak lungs, who had suffered from atony of the uterus after confinement with her sixth child, was brought to bed, for the seventh time, of a puny male infant, which, notwithstanding it was well fed, continued extremely emaciated. From its birth to its death, it frequently held its breath. This difficulty in breathing was at first hardly remarked, but it increased extremely, and came on particularly on waking, swallowing, or crying. It died in the seventh month, in a fit of suffocation with convulsions. An examination was not made, nor did the case make much impression on Dr. Kopp, until he had seen two similar cases in the children of the same mother.

CASE 2. A brother of the first infant, born at the full term, having a delicate constitution, but enjoying good health for the first four months, began from that period to be affected similarly, by a suspension of respiration at intervals, attended each time with a slight plaintive cry, coming on suddenly, and evidently producing pain. After gaining its breath, it testified by its cries the pain and anxiety it had suffered. During the paroxysm, the pulse was irregular and intermitting, eyes fixed, hands and feet cold, face puffed and bluish, and tongue pushed out between the teeth. The attacks increased in frequency and violence, but with this exception the health was not impaired. After an attack of catarrh when the child was ten months old, they increased greatly, and the child died in one of the paroxysms in a state of asphyxia; the face puffed and blue, and the tongue thrust between the teeth.

*Examination, twenty-two hours after death.* Tongue rather long, and thickened at the root; trachea healthy; thyroid gland tumefied; sanguineous extravasation covering the trachea at the junction of the thyroid and thymus glands. Thymus so large and thick, that an assistant took it for a lobe of the lung; extending from the thyroid gland to the diaphragm; two inches broad, weighing more than an ounce, and pressing against the trachea, where the effusion had taken place: on cutting it, much milky fluid, which had penetrated its whole substance, escaped; it was nowhere indurated. Lungs brownish red, gorged with blood, as in asphyxia. Heart flabby and atrophied. Foramen ovale open.

CASE 3. Twenty months after the death of the last child, the mother was confined with another boy, of a similar constitution, but rather stronger. Fifteen weeks after its birth, the same symptoms as in the other two cases commenced, so that the mother recognized the disease; there was also spasmodic contractions of the hands and feet. The treatment consisted in leeches to the epigastrium, calomel, lavements of valerian and musk, frictions with white precipitate, which caused an eruption followed by some amelioration. Under these means the nervous symptoms diminished, and, after taking six baths of infusion of chamomile and valerian, (which were discontinued, as they exhausted,) the paroxysms diminished in frequency, so that many days were passed without any. On examining the chest, the pulsation of the heart could not be discovered. Sometime afterwards, without any known cause, there were slight spasms of the hands and feet, with abdominal pain, contraction and slight swelling of the features, and protrusion of the tongue. Flowers of zinc, musk, ipecacuanha, and calomel, were of temporary benefit; and sometimes the paroxysm was shortened by laying the child on its face, and gently patting its back. The child however emaciated, its digestion was affected, and it vomited and had whitish and greenish stools; the paroxysms returned with violence, and in one of them it died asphyxiated, in the twenty-second week of its existence.

*Examination, twenty-seven hours after death.* Thymus very large, occupying the whole anterior part of the thorax, and adhering strongly to its superior part: it touched the thyroid gland, and covered the heart; it weighed an ounce; it was infiltrated with a milky fluid. Tongue large, thick, and protruded between the teeth. Glottis free; trachea healthy; heart flabby; foramen ovale closed; right lung gorged with blood; pulmonary tissue otherwise healthy, and floating in water. Cerebral substance slightly softened; abdominal viscera sound.

The two following cases were communicated to Dr. Kopp by Dr. Rullman, and they were both children of the same mother.

Case. The disease began during the third week, and the symptoms were exactly similar to those detailed by Dr. Kopp. In the twenty-first month, the child, whilst playing, stooped to pick up something he had dropped: he was immediately attacked with vertigo, and fell backwards into his father's arms; his face turned red and blue, his limbs became rigid, his skin pale, the stools and urine escaped involuntarily, and the child died suffocated. On dissection, it was found that the thymus extended from the upper border of the sternum to the diaphragm, covering not only the trachea and pericardium, but the whole anterior surface of the lungs. Its texture was rather firmer than usual, granulated, and infiltrated with a milky fluid. Lungs small, compressed, and gorged with blood, but otherwise healthy.

The other case was more fortunate. The same symptoms came on about the third month, and were aggravated at the sixth month, owing to dentition. As Dr. Rullman was aware of the cause, he adopted more suitable means: he prescribed Plummer's pill (in powder) with hemlock, applied a blister to the sternum, recommended low diet, fresh air, and the avoidance of any sources of disquietude. Gastric or catarrhal symptoms were carefully combated. During the first eight weeks there was no improvement, and the symptoms were aggravated whenever a tooth was cut: then purgative doses of calomel were useful. From this time the symptoms improved, and at the end of the second year the child was well. He is now nine years old, and quite healthy.

[The memoir of Dr. Hirsch contains five cases which are so similar to the foregoing ones, in every important particular, that we shall not give the details. The first was in a female infant, and no examination took place; the other four were boys. Two of these died, and the thymus was found enlarged; the other two lived. The treatment consisted in applying leeches and counter-irritants to the sternum, and giving calomel and rhubarb internally. In one case, small doses of Aqua lauri cerasi and musk were also prescribed. We shall now pass on to the general history of the disease, treatment, &c., as collected by Dr. Hirsch from his own cases and those of others.]

It is characterised by attacks of spasm of the chest and severe fits of suffocation. The breathing suddenly stops, or rather there is an extremely slight, piping, imperfect inspiration, forced, as it were, through the contracted glottis. The respiratory sound has some resemblance to the crowing inspiration of hooping-cough, but is much smaller and more acute; it is still more like the choking attempts at inspiration made during the hysteric spasm. In some cases, but rarely, there may be five or six piping or whistling inspirations, and then a few deeper and stronger, alternating with expirations so slight as scarcely to be perceived. In extreme attacks, the respiration stops *entirely*; the small inspiratory pipe then takes place, either in the beginning of the paroxysm or in its termination, being quite suppressed during the strength of the attack; and this symptom is pathognomic of the affection. The other symptoms are necessary consequences of the suppressed respiration; the body is extended forcibly backwards or the limbs drawn close up together; the face, which is fixed and expressive of great distress, is either purple or quite pale; the nostrils are expanded, the eyes fixed, the hands cold, the thumbs contracted, with involuntary dejections, &c. After a period of half a minute or a minute, occasionally even two or three minutes, the paroxysm ceases; the infant then cries for a short time, and immediately becomes cheerful and easy. When the constitution is very feeble, however, or the attack has been very severe, the child remains some time languid, pale, and sleepy. In the intervals of the paroxysms the child is quite well, without the slightest affection of the respiration. Kopp says that the tongue still continues to be thrust out more than is natural, but this is certainly not always the case. The attacks come on chiefly when the child awakes, at other times after crying or fretting, or from any cause that excites the lungs to increased action. At first the attacks are not frequent, coming only once in eight or more days; but they grow progressively more frequent, till they come on ten or twenty times in one day. In this stage, death often takes place in the paroxysm, although the little patient had been playing about a minute before, apparently in the most perfect health. More freqqently, however, another stage succeeds before death, characterised by general convulsions of an epileptic kind; the convulsions sometimes alternating with, sometimes accompanying, the asthmatic paroxysms. In the intervals, the lumbrical muscles of the palm and the adductors of the thumb are sometimes permanently contracted, so as to maintain a rounded form of the hand. In these cases the child is carried off in a paroxysm, with symptoms both of suffocation and apoplexy, or quite suddenly, without any affection of the chest or prolonged agony of any kind.

On examination, besides the general appearances always following death from suffocation,—such as blueness of the skin, congestions of blood in the lungs and brain, &c.,—the thymus gland is always found considerably enlarged. The degree of enlargement varies extremely, as does the natural size of the gland. At times it is enlarged chiefly in length and breadth, more commonly, however, in thickness: in the last case the lungs are frequently thrust back by it into the posterior part of the chest; on other occasions, the gland is found closely connected with, and even surrounding, the large arteries and venous trunks of the thorax or neck. The texture of the gland is either quite normal or (what is more common) somewhat firmer and more fleshy and redder, but without the least trace of induration, suppuration, tubercalization, or other form of degeneration. In cases where the gland has been weighed, it has been found to vary from six or seven up to fourteen drachms. However, it would seem to have been still larger in some cases where it was not weighed.

The duration of the asthma of Kopp is very various, depending generally on the severity of the attacks: it commonly lasts some months. Children of a scrofulous habit are particularly subject to it, and boys especially, if indeed not exclusively. All diseases of the respiratory organs predispose to it,—such as catarrh, bronchitis, croup, hooping-cough, measles. Teething also predisposes to it.

Thymic asthma is far from being a rare disease, and has been noticed by many writers, who have given it other names. The principal of these is Marsh, who describes it under the name of *spasm of the glottis*, Kellie, Porter, Pretty, Richter, and perhaps North. Even the enlargement of the thymus gland as a cause of asthma in children has been recognised by several authors previously to Kopp, and particularly by Hood. (*Edinb. Journ. of Med. Sc.*, vol. iii.)

*Nature of the Disease.* It is an affection peculiar to early infancy, and consists of a periodical tonic spasm of the lungs, larynx, and glottis, perhaps also of the heart, eventually extending to the whole nervous system, under the form of epileptic convulsions. In it the thymus gland is more or less enlarged, but otherwise not diseased, and presses on the heart, bronchi, and large vascular trunks, impeding their functions. When, on examination after death, such an enlargement and abnormal compression exist, one need not seek further for proofs of the nature of the disease. Yet there have been many arguments adduced against the accuracy of this judgment, of which the following are the principal:

1. Great enlargement of the thymus has been found, without the presence of asthma.
2. The affection termed the asthma of Kopp has been observed when the thymus was not enlarged.
3. An organic disease cannot occasion periodical attacks, leaving intervals of perfect health.
4. The enlarged thymus ought to excite symptoms of cardiac disease, while the suffocation in the disease of Kopp is evidently the result of constriction of the trachea and glottis.
5. The course, frequent cure, and occasional relief from antispasmodic remedies, are incompatible with the existence of an organic disease.
6. The enlarged thymus may be a consequence of the asthma, and not the cause.

*Prognosis.* The disease is always dangerous, but not desperate, particularly if the child is strong, and not disposed to catarrhal affections; also when the case is recent, the spasms not frequent nor severe, and no general convulsion has taken place.

*Treatment.* The following are the principal indications:

1. In the attack nothing more can be done than to place the child in a proper position, to rub its spine, perhaps to dash cold water on it, &c.
2. After the attack, to give antispasmodics, preparatory to the employment of means directed to the chief pathological state.
3. To diminish and prevent the recurrence of all undue congestion and nervous excitement in the heart and lungs, by low diet, large and frequent local bleedings (every four or eight days,) blisters and issues on the chest, constant powerful purgatives, &c.
4. To lessen the size of the thymus by anti-scrofulous, resolving medicines,—such as mercury, iodine, &c.

[We have been induced to present to our readers the foregoing account of the affection there denominated *Thymic Asthma*, with the view of calling the attention of the profession to a more accurate investigation of its nature and all its causes, and not because we subscribe to the general accuracy of the statements and opinions therein contained. It is hardly necessary to inform the English reader that the affection noticed in the present paper was long known in this country previously to the publication of Dr. Kopp. This is indeed admitted by Dr. Hirsch; and all pretence to originality in the views of Dr. Kopp respecting its cause is removed by the reference made by him to the paper of Mr. Hood, in the Edinburgh Journal of Medical Science, published in January, 1827. In this paper the existence of the enlarged thymus in such cases is proved by numerous dissections, and recognised by the author as a cause of the disease. The title of *Koppian Asthma*, bestowed on this disease by Dr. Hirsch, is evidently improper; and that of *Thymic Asthma*, given to it by Dr. Kopp himself, is no less so; since it can be demonstrated to have often existed without any morbid enlargement of the thymus gland. At the same time we do not doubt that such an enlargement is occasionally present, and operates as an exciting cause of the affection. The whole subject assumes at this moment a more striking interest from the publication of Dr. Ley's work on the same curious affection, and denominated by him *Laryngismus stridulus*, of which notice is taken in another part of this Number. We may refer our junior readers for a neat epitome of the history of this disease, with references to the principal original writers on it, to the article *Spasm of the Glottis*, by Dr. Joy, in the Cyclopædia of Practical Medicine. Dr. Joy, however, takes no notice of the *Thymic* theory of the disease, and refers neither to Mr. Hood nor Dr. Kopp.]

*Hufeland und Osann's Journ. der Prakt. Heilk., Jul.* 1835.

*Illustrations of the Diseases produced in Man by the Affection termed Glanders in Horses.* By Dr. ALEXANDER, of Utrecht; Dr. BERRA, of Mantua; and Dr. FENELLI, of Ostiani.

[ALTHOUGH the occurrence of morbid phænomena of the greatest severity in the human subject, from the contagion of Glanders, is no longer a novelty, we believe the following narratives will be found to contain matter that is both new and interesting; particularly the pestilential epidemic, for it may be so termed although on a small scale, recorded by the Italian physicians. We presume that no rational doubt can exist of the derivation of all the cases from the alleged source.]

I. *Cases of Glanders in Man.* By F. S. ALEXANDER, M.D.

CASE 1. A Lancer, aged forty, employed in taking care of a glandered horse, was brought on the 13th of April, 1829, to the hospital; he first declared himself ill on the 7th, but had not been well for some time previously. He complained of pain in the limbs; and, on examination, several unequal swellings were discovered, one on the fore-arm, another on the left knee, a third on the calf of the left leg, which rendered motion painful, were elastic to the touch, and of the natural colour of the skin.

In the beginning of May the patient continued to lose flesh, and had night sweats; the gums bled; the swelling on the arm diminished; the others remaining stationary. In the beginning of June a mercurial liniment was rubbed over the tumours; some of which diminished, others disappeared: one on the leg, however, enlarged, grew painful, and was opened on the 11th with caustic; bloody pus was discharged. The cough grew more severe in August, and there was constant diarrhœa; on the 16th of that month the patient died, much emaciated.

*Examination forty-eight hours after Death.* The openings of the abscess were found extending round the muscles; the cellular substance was destroyed completely; the muscular fibres remained, although wasted and of a yellow colour; the matter in the tumours was so viscous that it did not escape; in the arm, no trace of the former tumour was found. The lungs were found adhering to the costal pleura; some parts were hepatized; others contained tubercles, crude, softened, or purulent. The mucous membrane of the trachea was inflamed; the heart appeared healthy; the intestines in the normal state, neither inflamed nor ulcerated. The brain and spinal marrow were not examined.

CASE 2. An artillery man, nineteen years old, after he had assisted in the infirmary for horses for four weeks, was seized suddenly with fever, violent stitches in the side, and pains of the lower extremities. In five or six days the fever and stitches left him, but the pains in the legs continued. On the 4th of June, 1831, he entered the hospital. On the 11th, a painful swelling arose on the calf of the left leg; on the 16th, a similar tumour appeared near the elbow of the forearm.—17th. Two more tumours were perceived on the leg and arm; the first tumour on the arm became of a violet colour, soft, and somewhat fluctuating. The right eyelid, swelled and inflamed, was opened with difficulty.—20th. Solitary pustules arose on the forehead, soon ran into each other, and were hid by a black scab covered with pus, which escaped from the skin in several places. Dr. Alexander now ascertained that some of the horses in the infirmary where the patient had been had glanders.—24th. The erysipelatous inflammation of the face spread, and the left eyelid was swollen, œdematous, and closed.—25th. The inflammation and pustules extended to the left cheek; the alæ nasi were covered with a dirty green, viscid mucus. The patient complained of difficulty of swallowing.—28th. The pulse was quick and small; respiration regular; the forehead blue; the patient was sleepy.—February 1st. Delirium; the tongue was tremulous, and put out with difficulty; there was intolerable thirst; the urine and stools passed involuntarily; the nose and left cheek were black.—Feb. 2d. The patient lay unconscious, swallowing with difficulty. He died at ten o'clock in the evening. Duration of the disease, eight months.

*Examination on the day after Death.* Several parts of the body were found covered with bullæ, filled with a bloody fluid. The lungs were adherent in many points; their surface was everywhere, with the exception of the superior lobes, spotted with tubercles softened in the centre; in the substance of the lungs, however, no tubercles were discovered. The trachea and bronchi were filled with a tough mucus; the blood in the aorta was coagulated. Solitary red spots were visible in the œsophagus, stomach, and intestines. The mucous membrane of the nose was ulcerated in many places, and covered with tough mucus. The velum palatinum, the bronchi, and epiglottis, were in part destroyed and covered with ulcers and mucus. The salivary duct was inflamed, thickened, and filled with pus; ulcers were disseminated over the larynx. Twigs of the facial and frontal nerves were inflamed; the temporal artery passed uninjured through the gangrenous parts. The brain was in the natural state. On dissecting the extremities, small purulent tubercles were found in the muscular as well as the cellular substance. The nerves were in the natural state, except where they passed through abscesses; the veins contained no pus; the lymphatics were dilated; the glands swollen; the fibula and ulna were denuded in several places.

Dr. Alexander infers that slighter cases of glanders in the human subject may be overlooked, from the circumstance that he treated another soldier of the same corps, about the same time, in whom the disease at first seemed likely to pursue a similar course: its violence, however, abated, without developing any local affection; it became lingering, and took on the form of an irregular nervous fever. After remaining dangerously ill seventy-two days, he however recovered, probably from possessing a strong constitution. In another case, a red spot appeared on the forearm of an artillery man, who had previously been cleaning a horse that had glanders; pustules broke out, and an abscess was formed, which, when opened, discharged pus. This did not cicatrize, but left a spreading ulcer, the edges of which, when the man came, on the 18th of June, to the hospital, appeared callous; a tough, ash-coloured, caseous pus was secreted. The ulcer healed in April, 1833, the thirteenth month of the disease.

*Hufeland und Osann's Journal,* 1835, B. 2, h. 2.

### II. *Carbuncular Typhus, occasioned by the Glanders in the Horse.*

*First Series of Cases.* By Giuseppe Berra, m.d.

Luigi Carnalengi, a miller of San Benedetto in Mantua, had in his stable two glandered horses, and his brother Martin had a mare, a mule, and an ass, affected with the same disease. The first person attacked was Natali, son of Martin, who took care of the three glandered animals, and slept near them during the whole of the spring. His illness began about the middle of March, and he died towards the latter end of the month. Andrew, son of Luigi, æt. six, who used to play in the stable and wipe the noses of the horses, was taken ill in the middle of May, and died on the 7th June. While Natali was ill, Martin, æt. fifty-eight, did his work for him, and experienced the same effects. He sickened June 8th, and died in ten days. Charles, son of Martin, æt. ten, who had long assisted his father and brothers, was seized June 2, and died on the 30th.

The disease began in all with either a slight intermittent quotidian fever, or with a tertian with gastric symptoms: next came on pains in the joints with a rather violent continued fever; the pulse hard and full, and all the symptoms of a rheumatic attack. After some days an extraordinary calm succeeded, followed however in a day or two by an exacerbation of all the symptoms, as hard pulse, flushed face, foul tongue, open bowels, hot skin, scanty urine, and a fixed and oppressive pain in the chest. The blood exhibited a remarkably hard clot, without the bleeding being followed by any good result. The fever became more ardent with the progress of the disease, and was attended with delirium, tremors, convulsions, meteorism, difficult deglutition, very dry tongue, and grave cerebral symptoms, with an almost continual stupor.

At the same time that the remission of the fever took place, very minute red spots appeared upon the face, soon surmounted by a white head, which passed rapidly into pustules of varied figure and dimensions, some remaining as small as millet seed, others with their areola becoming as large as a split walnut, and all containing a true pus. They did not all burst, and those that opened spontaneously after a longer or shorter time, left deep foul ulcers of a violet colour at their bottom. This did not occur in those that were opened accidentally or with the scalpel. The pustules were of a lead colour and contained a yellowish fluid, inodorous but particularly acrid. In one case they occurred more particularly upon the face, of the size of nuts, confluent, and leaving, after they had burst, an ulcer extending from the forehead to the zygoma and upper lip. Besides these pustules, red scattered spots of different sizes appeared, similar to phlegmonous erysipelas, which remained in the same state without proceeding to suppuration. With these typhoid and exanthematous symptoms, the disease advanced, till the patient, having lost his external senses at the end of the second or third week, died after a most painful and distressing struggle.

*Second Series of Cases.* By Signor FENELLI.

The disease exhibited two distinct periods; one marked by simple fever, the other by the eruption. The precursory symptoms were universal muscular pains, and failing of appetite, sleep, and strength, without any loss of spirits. The attendant symptoms were rigors, pains as if the individual had been beaten, oppressive headach, heat and dryness of skin, mucous crust upon the tongue, urine red, pulse rapid, full, and regular, blood thick and buffed.

After the seventh day, the second period came on with diminished fever, but with great oppression of the chest, delirium, and restlessness at intervals, and partial sweats. The face of all the patients was drawn, and their urine deposited a sediment; but there never was any epistaxis. On the backs of the hands and feet a red swelling arose, attended with lancinating pains, and terminating in gangrene and sphacelus. Besides these parts, others were similarly attacked by gangrene, particularly the face. After the falling of the eschar, an unhealthy ulcer, distilling a fœtid ichor, was left in those who survived up to this time. Besides the gangrene, purulent phlyctenæ, pustules, and papulæ, arose on different parts of the body; but in one woman, who died on the sixteenth day, nothing but livid marks were observed.

The disease lasted from nine to twenty days, with a constant decrease of the strength and great præcordial anxiety, till the patients died in a state of stupor. One woman died thus after the fiftieth day, and another after the sixtieth, in whom, after an apparent healing of the ulcers, a general eruption broke out like scarlatina, followed by another crop of carbuncles. Every case being fatal, the treatment does not admit of remark. No examination of the bodies was made, from fear of contagion; but the disorder was confined to twelve persons who had attended upon diseased horses, and none of those who waited on the sick were attacked; although the curate, who attended them all in their last moments, had an attack somewhat similar, from which however he recovered.

*Antologia Medica*, No. ix. *Settembre*, 1824.

---

*New Mode of administering Balsam of Copaiba.* By M. RATIER.

M. RATIER is now in the constant habit of prescribing balsam of copaiba for gonorrhœa, enclosed in small capsules made of gelatin. They each contain eighteen grains of the balsam, and are sufficiently thick to prevent their breaking in the mouth, or giving a disagreeable taste, and yet readily soluble; whilst, from their small size and oval shape, they are swallowed with facility. M. Ratier generally directs half the number prescribed daily for internal use, to be introduced into the rectum, which is easily done if they are covered with oil or suet. As mentioned in our last Number, M. R. has been long in the habit of directing his patients to throw into the rectum, by means of a small syringe, one or two teaspoonfuls of the copaiba emulsion, at the same time as he ordered it to be taken by the mouth; but these capsules are in all respects preferable. They were invented by MM. Dublanc, Sr. and Mothes, and are formed by a particular instrument.

*Bulletin Général de Thérapeutique, Decembre*, 1835.

---

*On the Effects of Indigo in Epilepsy and other Spasmodic Affections.*
By Dr. Roth, of Mayence.

A Number of the *Salzburg Med. Chir. Zeitung*, for 1832, contains a paper on this subject by Prof. v. Leuhossek, wherein it is stated that Prof. v. Stahly, of Buda, had been the first to administer indigo in spasmodic affections, and that the successful results had been noticed in an inaugural dissertation on epilepsy, which appeared at Buda in 1832. This was followed in 1833 by an article in the "Med. Zeitung" by Dr. Grosheim of Berlin, who, it appeared, had employed indigo with entire success in an epileptic case which had previously resisted every other kind of treatment.

These precedents were the occasion of a series of trials being instituted at the Charité at Berlin, by Dr. Ideler, superintending physician of the wards for spasm and insanity, and by Prof. Wolff, chief physician to the other medical wards, as well as by Dr. Leinveber, one of the staff physicians at the Charité.

Although in the selection of cases attention was paid to the remark of Leuhossek that indigo only acted beneficially in the epilepsy directly depending upon abnormal sensibility and reaction of the nervous system, (abnormer sensibilität und reaction,) the remedy was nevertheless tried on a considerable number of patients; it being frequently difficult to decide whether epilepsy is idiopathic or merely symptomatic, and it also often happening that cases originating as the latter degenerate into the former. At the period when these experiments were made, the greater number of epileptic cases at the Charité differed from each other only in their degree of intensity, and the seat of the affection appeared to be either the brain itself or the cerebral nerves. In no instance did the spinal cord seem to be the chief seat of the morbid action. Most of the patients had already gone through the ordeal of the most powerful remedies without material benefit. At the same time subjects whose epileptic attacks were the consequence of external injury of the head, such as contusions, &c. were not excluded from the trials.

Dr. Roth had an opportunity of witnessing the experiments during a period of eight months, and the following are the general results obtained by him:—The remedy is beneficial in all cases of true *idiopathic* epilepsy, curing those of recent origin and improving [i. e. modifying both the violence and the frequency of the paroxysms] those of long standing. On the other hand few cases of *symptomatic* epilepsy were ameliorated—none cured,—by this treatment.

*Of the Effects of Indigo on the System.* Prof. v. Stahly appears to have been led to the administration of indigo from having observed that the hands of *sickly* indigo-dyers were permanently tinged with blue, which was not the case with dyers in full health. During the first days of its employment, says Dr. G. v. Stahly, jun.,* "the patients are affected with a slight diarrhœa, which however ceases spontaneously when the indigo begins to be more easily assimilated with the digestive organs. Even from very large doses, to the amount of two ounces, no other visible effect is produced beyond that of the urine and perspiration assuming a blue colour." Dr. Roth however affirms, that with the greater number of patients very different phænomena are observable. In most instances a sense of constriction at the fauces and the impression of a metallic taste on the tongue shortly succeed the first administration of indigo, although the medicine itself is perfectly tasteless. Nausea, inclination to vomit, &c., with delicate persons even vomiting, follow in succession: at times the retching is so violent as to preclude the further use of the remedy, whilst in the other cases the nausea continues for two or three days, and then gradually subsides. The retching and vomiting produced by indigo differs somewhat from those which are the effect of common emetics; the contractions of the abdominal muscles and of the diaphragm being less violent, and the act not being succeeded by the usual feeling of distress and prostration. The egesta present no peculiarity except in their blue colour. After all inclination to vomit has ceased, a diarrhœa usually takes its place, increases rather than diminishes as the treatment proceeds, and lasts during the whole time that the indigo continues to be administered. Thus from four to six stools of a semifluid consistency and a blue-

* In the dissertation already alluded to.

black colour are produced daily, with the accompaniment of slight gastro-enteric pains, which are likewise observed during the period of nausea and retching, and are in rare instances so violent as to proscribe the further use of the remedy. The protracted diarrhœa ultimately brings on dyspeptic symptoms with vertigo, &c. In one or two instances, the bowels remained obstinately constipated instead of being relaxed. Indigo does not appear to increase the quantity of the urine, but invariably to tinge the latter of a dark violet hue, paler in the urine secreted at night than in that of the morning.

Dr. Roth has never found that indigo affected the colour of the perspiratory matter. Slight spasm and twitchings of the tendons sometimes arose after the treatment had lasted for a few weeks.

*Form of Exhibition.* The experiments were made with the Guatimala Indigo of commerce [from the Indigofera Disperma, or the Argentea.] The best mode of administering it is in the form of an electuary, in which one portion of indigo is combined with two of syrup and a small proportion of water. In the form of powder it is apt to occasion distressing spasm at the isthmus faucium. Its tendency to produce diarrhœa may be corrected by the simultaneous use of mild tonics or astringents. The pulv. ipecacuanhæ comp. was found to answer this purpose sufficiently well.

*Dose.* Small doses appear to be wholly ineffectual. If the remedy agrees with a patient at all, it is necessary to commence with tolerably large doses, which may be gradually augmented to an ounce or even more, according to circumstances, per diem; the corrigents to be increased in the same proportion. The treatment it may be necessary to continue for three or even more months.

*Effect on Epileptic Patients at the Charité.* At the commencement of the treatment, the frequency of the paroxysms was in every instance manifestly increased. Each attack was of shorter duration than formerly, but more violent; the subsequent prostration therefore more considerable. The premonitory and soporous stages were less prolonged than usually.

After from one to eight weeks, according to the dose administered, all the above phænomena gradually began to abate. In the successful cases the paroxysms diminished in frequency, duration, and violence, and the last attacks amounted to no more than slight shiverings and inconsiderable twitchings.

The total number of cases of epilepsy treated with the indigo was twenty-six. It has already been stated that only those were cured by it whose epilepsy was idiopathic. The number of cures witnessed by Dr. Roth amounted to nine males and five females. Eleven others were relieved, whilst in six cases the complaint underwent no improvement. Dr. R. was particularly struck with the instance of a boy, aged sixteen, who for eight years had been the victim of chorea St. Viti, to which epilepsy had supervened, but who was radically cured of this double affection by a six-weeks' use of indigo.

*Hecker's Neue Annalen. Erster Band. Erstes Heft.* Berlin, 1835.

---

*Case of unusually prolonged Sneezing, cured by Snuff.* By Dr. Bauwens.

[The following case was read before the Society of Medical Sciences of Brussels, and is remarkable no less for its severity than for the simplicity and speediness of the cure. If homœopathy could adduce many such marked illustrations of the power of remedies administered according to its formulæ, and on the principle acknowledged long before it, as occasionally a faithful guide in practice—*similia similibus curantur,*—it would meet with less opposition from the enlightened members of our profession.]

Caroline D. æt. eleven and a half, was attacked, on November 18th, 1834, with fits of sneezing, which gradually increased in frequency, so that they came on every quarter of an hour, and continued until the 8th of January, 1835, when Dr. Bauwens first saw her. Although delicate, yet she had always been healthy, and all her functions were natural, except sleep, which was interrupted by the sneezing. This she attributed to a tickling sensation in the nasal mucous membrane, and she complained during the fits of vertigo and headach. On examining the nostrils, no trace of inflammation or any foreign body could be discovered. A detailed account follows of the various measures which were adopted from this date to the 21st of March. These were fomentations and baths; then purgatives and foot-baths; next low diet; anthelmintics; sulphate of quinine in large doses; emetics; antispasmodics; and homœopathic doses of nux vomica; but no relief was obtained: at last, Dr. Bauwens prescribed snuff. Three pinches, given at a few hours' interval, produced at the time violent sneezing and some mucous discharge from the nostrils, but entirely cured the patient.

*Bulletin Médical Belge. No.* 1. *Janvier,* 1836.

---

### *The Magnet, a Remedy for Gout.*

Owing to a considerable commercial demand for loadstone, the conductors of the *Bulletino delle Scienze Mediche* of Bologna were led to make enquiries concerning the uses to which it was put; from which it appears that the Ex-Bey of Algiers, whilst at Leghorn, in 1831, mentioned to a Catholic dignitary, (Father Campagnoli,) who was suffering from the gout, that the application of the loadstone was an oriental remedy for that disease, and of certain efficacy. The patient immediately procured a piece of loadstone, as he had been subject to regular and frequent attacks of gout since 1805, and the application removed the next paroxysm. Since this time he has always recurred to the same remedy, and he finds that the attacks came on less frequently and severely, and that they invariably yield, so that he has rejected all his former plans of treatment. On the first symptom he goes to bed, and places the loadstone in close contact with the painful part; he presently falls asleep, and awakes free from pain and able to walk. The loadstone he uses weighs five pounds and has smooth sides. He has recommended to other gouty persons the same remedy, and they have experienced similar relief.—*Bullettino delle Scienze Mediche. Bologna. Marzo et Aprile,* 1835.

---

### *On the Relations between the State of the Pulse, Respiration, and Temperature of the Body, in Diseases.* By Dr. Al. Donné.

[The want of accurate information on these points led Dr. Donné to direct his attention to them, whilst acting as "Interne" at the hospitals of la Pitié and la Charité; and, as he has no longer the same opportunities of pursuing his investigations, he has arranged the facts he has collected in tables. Although the inferences he draws from them are but few, and the data not sufficiently extensive, he hopes that he may be the means of directing the attention of others to the same subject. The temperature was taken by a small thermometer placed in the armpit, and kept there for five or ten minutes. To have placed it in the mouth or anus would have been impracticable, and, on a comparative trial of the heat of the mouth and armpit, no marked difference was detected.]

| | Pulse. | | Respiration. | | Temperature.* |
|---|---|---|---|---|---|
| Hypertrophy of the heart.... | 150 | .. | 34 | .. | 103 |
| Puerperal fever (Metritis) .. | 168 | .. | 48 | .. | 104 |
| Phthisis.................. | 140 | .. | 62 | .. | 102 |
| Typhoid fever ............ | 136 | .. | 50 | .. | 104 |
| Jaundice ............... | 36 | .. | 37 | .. | 96.40 |
| Hypertrophy of the heart.... | .. | .. | .. | .. | 94.40 |
| Diabetes ............... | .. | .. | .. | .. | 96.40 |
| Lumbago ............... | .. | .. | 16 | .. | .. |

The preceding cases are those in which the temperature observed was the highest and lowest, and the respiration and pulse most frequent and slowest. It will be observed that, in some of the cases, the temperature was in relation to the rapidity of the pulse and the respiration; but the relations of these three conditions to each other are far from being exact.

* Fahrenheit's scale is used throughout this paper.

*Pulmonary Tubercles.*

*Women.*

| | Pulse. | Temperature. |
|---|---|---|
| First case.... | 120 | 102.10 |
| | 128 | 99.50 |
| | 120 | 102.10 |
| | 112 | 100.20 |
| | 136 | 99.50 |
| | 126 | 99.20 |
| | 130 | 100.20 |
| | 140 | 102.10 |
| Second case.. | 82 | 99.50 |
| | 76 | 99.50 |
| | 84 | 98.30 |
| Third case .. | 88 | 102.10 |
| | 96 | 100.20 |
| | 76 | 101.30 |
| | 106 | 101.30 |
| | 98 | 100.20 |
| | 98 | 99.50 |
| Fourth case.. | 100 | 101.30 |
| | 104 | 100.20 |
| | 110 | 102.10 |
| Fifth case .. | 128 | 101.75 |
| | 120 | 101.75 |
| | 112 | 100.85 |
| | 100 | 100.20 |
| | 84 | 98.30 |
| Sixth case .. | 68 | 96 |
| | 92 | 103 |
| | 74 | 98.30 |
| *Men.* | | |
| Seventh case, | 126 | 99.50 |
| | 116 | 98 |
| Eighth case .. | 87 | 99.50 |
| | 62 | 98.30 |
| | 72 | 100.20 |
| | 80 | 101.30 |
| | 88 | 102.10 |
| | 78 | 100 |
| Ninth case .. | 104 | 101.50 |
| | 104 | 100 |
| | 100 | 100.20 |
| | 110 | 100.20 |
| Tenth case .. | 76 | 99.50 |
| | 80 | 97.30 |

In some of these cases there is a correspondence between the state of the pulse and temperature. There is not, indeed, an exact correspondence between the number of pulsations and the degrees of the thermometer; but the heat of the body augments at the same time as the pulse quickens, and *vice versâ.* This occurred in Cases 5, 6, 7, and 8; but, in Cases 1, 2, 3, 4, 9, and 10, there is no relation; for in some the temperature diminishes when the number of pulsations increases, and in others the heat increases whilst the pulse becomes slower.

*Pleurisy.*

*Men.*

| | Pulse. | Temperature. |
|---|---|---|
| First case.... | 106 | 99 |
| | 104 | 100.20 |
| | 106 | 100.20 |
| Second case.. | 80 | 94.38 |
| | 80 | 100 |
| Third case .. | 88 | 98 |
| | 80 | 99 |
| | 94 | 98.30 |
| Fourth case.. | 100 | 101.30 |
| | 100 | 102.10 |

*Pneumonia.*

| | Pulse. | Temperature. |
|---|---|---|
| First case.... | 102 | 101.30 |
| *(Woman.)* | 90 | 99.50 |
| | 94 | 100.20 |
| | 98 | 101.30 |
| | 86 | 101.30 |
| Second case.. | 92 | 96.40 |
| *(Man.)* | 126 | 103 |

*Hypertrophy of the Heart.*

*Women.*

| | Pulse. | Temperature. |
|---|---|---|
| First case.... | 61 | 97.75 |
| | 68 | 98.30 |
| Second case.. | 120 | 100 |
| | 120 | 101 |
| Third case .. | 64 | 98.30 |
| | 98 | 99.50 |
| Fourth case.. | 106 | 99.50 |
| | 108 | 102.10 |
| | 106 | 101.58 |
| | 108 | 99.72 |
| | 104 | 99.50 |
| | 150 | 103.10 |

### *Chlorosis.*

| | Pulse. | Temperature. | | Pulse. | Temperature. |
|---|---|---|---|---|---|
| First case.. . | 92 | 100.2 | | 100 | 98.30 |
| | 80 | 97.75 | | 82 | 98.30 |
| | 74 | 96.40 | | 96 | 99.72 |
| | 74 | 99 | Third case .. | 104 | 99.50 |
| Second case.. | 72 | 99 | | 102 | 100.20 |
| | 96 | 99 | Fourth case.. | 108 | 100.20 |
| | 100 | 99.50 | | 100 | 101.75 |

### *Metro-peritonitis (Puerperal Fever.)*

| | Pulse. | Temperature. | | Pulse. | Temperature. |
|---|---|---|---|---|---|
| First case.... | 152 | 102.10 | | 157 | 103.55 |
| | 156 | 102.10 | | 166 | 103.10 |
| | 168 | 104 | | 152 | 102.10 |
| | 152 | 103 | | | |
| | 154 | 103.55 | Second case.. | 96 | 99·50 |
| | 152 | 103.55 | | 104 | 100 |

### *Jaundice.*

| | Pulse. | Temperature. | | Pulse. | Temperature. |
|---|---|---|---|---|---|
| First case.... | 36 | 98 | Second case.. | 62 | 97.75 |
| *(Man.)* | 38 | 98.30 | *(Female)* | 52 | 96.40 |

### *Hemiplegia.*

| Pulse. | Temperature. | Pulse. | Temperature. |
|---|---|---|---|
| 124 | 99.72 | 96 | 99.50 |

### *Hysteria.*

| Pulse. | Temperature. | Pulse. | Temperature. |
|---|---|---|---|
| 76 | 99.19 | 94 | 98 |
| 94 | 98 | 102 | 99.72 |

### *Diabetes.*

| | Pulse. | Temperature. | Pulse. | Temperature. |
|---|---|---|---|---|
| *(Man.)* | 78 | 97.25 | 84 | 96.40 |
| | 78 | 97.25 | | |

### *Enteritis.*

| | | Pulse. | Temperature. |
|---|---|---|---|
| *(Man.)* | Before bleeding .... | 104 | 101.58 |
| | After bleeding...... | 76 | 100.20 |

### *Rheumatism.*

| | Pulse. | Temperature. | | Pulse. | Temperature. |
|---|---|---|---|---|---|
| First case.... | 86 | 98.30 | Third case .. | 76 | 98.30 |
| *(Man.)* | 82 | 98.30 | *(Man)* | 60 | 97.39 |
| Second case.. | 96 | 101.75 | | | |
| *(Woman)* | 80 | 99.50 | | | |

### *Inflammatory Fever.*

| | Pulse. | Temperature. | | Pulse. | Temperature. |
|---|---|---|---|---|---|
| First case.... | 102 | 104 | Second case.. | 72 | 97.75 |
| | 72 | 97.75 | | 88 | 101.30 |
| | 60 | 96.40 | | | |

### *Typhoid Fever.*

| | Pulse. | Temperature. | | Pulse. | Temperature. |
|---|---|---|---|---|---|
| First case.... | 88 | 101.30 | Second case.. | 102 | 101.30 |
| *(Man,)* | 90 | 102.10 | *(Woman.)* | 108 | 103.55 |
| | 84 | 101.30 | | 136 | 104 |

From the foregoing observations it appears that a relation between the temperature and pulse existed more frequently than the contrary, especially in those diseases which did not interfere considerably with the functions of hematosis. Thus, in tubercular consumption, pleurisy, and chlorosis, there was a more frequent want of relation than there was relation; in organic diseases of the heart, and contraction of its orifices, the relation was by no means constant; in hemiplegia, enteritis, rheumatism, jaundice, inflammatory, intermittent, typhoid, and puerperal fever, the relation was more constant. The constancy in pneumonia was an exception to the other pulmonary diseases.

[Two tables of the state of the pulse and respiration, and of the respiration and temperature, conclude this paper. M. Donné avoids drawing any inferences from these, or attaching much importance to his other deductions, as his facts are not sufficiently numerous: as far however as they go, they are of importance; and, if M. Donné succeeds in inducing others to take similar pains, sufficient data may be eventually obtained to elucidate that very obscure subject, the production of heat.]

*Archives générales de Médecine, Octobre*, 1835. Tome lx.

---

*On the Presence of Worms in the Air-Passages.* By L. ABOUSSOHN.

[THIS cause of death has attracted but little attention. In 1822, when M. Aboussohn first met with the following case, he could only find one other recorded, which was by Haller, of a girl of ten years old, who died from suffocation produced by two lumbrici in the trachea. (*Opuscula Pathologica.* Lausanne, 1768, p. 26, *Obs.* 10.)]

CASE. B. R., a little girl of nine years old, and of good constitution, was seized, on 30th December, 1822, on going to school, with difficulty of breathing, which was attributed to the violence of the wind. The dyspnœa increased, and in the night she could not lie down, and often ground her teeth. To these symptoms were added, on the next day, copious perspirations, from constant and uncontrollable agitation; there was some vomiting. She swallowed with great difficulty a few spoonsful of infusion of valerian, given by her medical attendant, who suspected hydrophobia, from her having been severely bitten by a dog forty-six days previously. She expected to die. On the morning of the third day she began to spit constantly, and referred all her distress to the anterior part of the chest. She drank at noon some sugared water, which she vomited with some relief: she afterwards requested food, which was also vomited. A general tremor of the whole limbs came on, followed by convulsions, trismus, and death at half an hour after one o'clock, in the midst of the most dreadful sufferings. The body was examined most carefully forty-eight hours after death, without any thing being discovered unnatural, with the exception of thirty-seven ascarides lumbricoides; one of which, five inches in length, was found partly in the trachea and partly in the right bronchus, of which the mucous lining was injected and covered with reddish mucus. The stomach contained two worms, the duodenum eight, and the jejunum twenty-six.

In 1834, M. Blandin mentioned, in his Traité d'Anatomie Topographique, (p. 199,) that, whilst at the Children's Hospital, he had met with a case of a child strangled by a large ascaris lumbricoides, which had ascended from the stomach, and had found its way into the trachea and right bronchus. The following case occurred in the same hospital.

CASE. A little girl, æt. 9, having suffered for two days the precursory symptoms of measles, was attacked suddenly with extreme anxiety, great dyspnœa, acute pain in the throat, to which she often carried her hand, as if to remove the obstacle, unsuccessful efforts to cough, threatening asphyxia: death, two hours after admission. On examination twenty-four hours after death, a worm, six inches long, was found alive in the pharynx, into which it had probably crawled from the larynx after the death of the patient. No other diseased appearances were discoverable, except some redness of the mucous coat of the stomach, and twenty lumbrici in the intestines. Although the mucous lining of the larynx and trachea was quite pale, and the worm was not found there, yet the sudden symptoms unexplained by any organic change, and the proximity of the worm, render it highly probable that its presence in the larynx was the cause of death. (*Bulletin général de Thérapeutique*, tom. viii. p. 32.)

The following case was more favorable. A young lady, æt. 8, whilst in good health, was suddenly seized, without any known cause, with cough, which in a few instants became very violent, with symptoms of suffocation. This state lasted two hours, and she became convulsed, when in her efforts she threw up a living worm (Acaris L.), and the cough and other symptoms ceased.

Case. G. Sweig, æt. 52, a labourer, habitually asthmatic, but otherwise in good health, was, after much anxiety and exhaustion in nursing his sick and poor family, seized with febrile symptoms, attended with dyspnœa and great prostration of strength. On the fifth day of the disease the dyspnœa increased, with so much agitation that the patient required several persons to keep him in bed. On the sixth day this continued, with threatening suffocation, whistling respiration, deglutition difficult; he could not speak, but by signs referred the seat of his suffering to the upper part of the sternum. He died suddenly in the afternoon.

*Examination, forty-two hours after death.* Abdominal and thoracic viscera were sound, but a lumbricus was found lying across the bifurcation of the trachea, mucous membrane injected, and in one part a superficial erosion. Probably this worm passed into the trachea on the fifth day of the disease, when the habitual dyspnœa became so unusually augmented.

Of these six cases, there were five in which the accident happened to children between eight and nine years old, four of whom were girls: the sixth case, however, proved that it may happen in advanced years, when circumstances are present which are favorable to the development of worms. The symptoms differ according to the situation of the worm: when it was in the larynx, the violent paroxysms of cough threatened suffocation; when in the trachea, the cough was less intense, there was rather dyspnœa with paroxysms of orthopnœa, and great agitation, vomiting, and incontinence of urine; death was preceded by convulsions in one case, and in the last was sudden, as if the lung, fatigued by the struggle, was deprived of all nervous influence. The impression of the patient, that a fixed and local obstacle prevents his respiration, is a valuable diagnostic sign. The means to be employed would be, 1, immediately to pass the finger into the throat, to examine if a worm can be felt, and to remove it. 2. Vomiting should be excited. 3. As a last resource, tracheotomy should be performed.

[We would suggest that, in those cases where the worm passes into the trachea, considerable light might be thrown on the diagnosis by the stethoscope. From the anatomical disposition of the right bifurcation of the trachea, foreign bodies pass into it more readily than into the left: in two of the above cases this happened; and of the other two, one is deficient in detail on this point, and in the other the worm is said to have lain across the bifurcation. Diminution or abolition of the respiratory murmur, *particularly in the right lung*, with absence of any physical signs of disease adequate to the explanation of the dyspnœa, when viewed in connexion with the history of the case and the symptoms, would render the diagnosis more certain. Indeed, the patient's life depends on the accuracy of the diagnosis, as probably tracheotomy alone would enable the worm to be expelled.]

*Archives générales de Médecine, Janvier*, 1836.

---

### *Salutary Effects of Smallpox.* By L. Polletti, m.d.

This case is related by Professor Lionello Polletti, of Ferrara. A girl had for a considerable time complete loss of sense and motion of the right leg, with permanent contraction of the right arm, which had been succeeded by what has been called paralysis agitans; all the usual remedies had been tried, but without success, so that she refused to submit to more, when she was seized (although previously vaccinated,) with confluent smallpox. She recovered, however, from the attack, which was very severe and alarming in all its stages, and at the same time lost the paralysis of the arm, and regained the motion and sensation of her leg.—*Bullettino delle Scienze Mediche. Bologna. Febbrajo*, 1835.

*Report on Vaccination in France during* 1833. By M. GERARDIN.

(*Experiments on the Nature and Reproduction of the Vaccine Virus.*)

THE numerous cases of modified small-pox which occurred in 1825 in France, in individuals regularly vaccinated, led to the supposition that vaccine matter had degenerated by successive transmissions for a long period. M. Fiard, by a long course of careful experiments, endeavoured to settle this point by ascertaining whether or not vaccine virus could now be transmitted from man to cows, and again from cows to man, as it could be at the commencement of this century when it was first introduced into France, as is proved by a report on the state of vaccination, made at that time. If this was not now possible the inference was legitimate that a change in the nature of the virus had taken place. In 1824 and 1825 M. Fiard introduced vaccine matter into seventy cows of different kinds; in only seven animals were pustules produced; the eruption was less developed than in the most feeble vaccinations, and the matter from the eruption introduced into children produced no effect. In 1828 M. Fiard received from England four glasses charged with vaccine matter taken from cows: with this he immediately vaccinated a young cow and the regular eruption followed. He then vaccinated eight children, and in two only was it unsuccessful, one of these having been vaccinated several times with ordinary matter. The eruption differed from common cowpox in the greater severity of both local and general symptoms. Altogether he vaccinated twenty-four children successfully from this same cow. The cow-pox of the inoculated cows was not attended as in the natural disease, as described in this country, by fever, loss of appetite, depression, and diminished secretion of milk. Eventually, from the difficulties attending the transmission of the virus, M. Fiard lost his cow-pox matter. His next endeavours were to ascertain if cow-pox existed among cows in France. He was then living in the department of Ain, and he was assured by the country people that such a disease was common among cows, rendering milking difficult, and that the women who milked the diseased animals always washed their hands in order that they should not communicate the infection to healthy cows. A cow was soon brought to him with several pustules like those of cow-pox on the teats. He inoculated five children with matter taken from these pustules without effect. Twelve days afterwards, on examining the same cow, he found new pustules springing up as the old ones dried up. These circumstances made him doubt the nature of the eruption. Similar experiments were repeated in this district many times in three successive years. In 1827 he repeated them near Paris, and at La Chapelle in 1828, with the same want of success; and he concluded that "it was impossible to procure the cow-pox in France." The information he received from other parts of England confirms a statement of Dr. Baron to M. Valentin as to the rareness of cow-pox in Gloucestershire: during fifteen years it has only appeared at Berkley in 1818, 1819.

[M. Fiard is by no means justified in inferring from his own researches that cow-pox matter cannot be procured in France. His search was continued only a few years, and in two or three districts. As it appeared in Berkley, a village in England, only twice in fifteen years, M. Fiard's experiments are certainly too few to admit his sweeping conclusion.*]

M. Fiard instituted the following experiments in order to determine whether (as Jenner thought) vaccine matter came from the disease of horses called, in France, *water in the legs*, in England, *grease;* or whether cow-pox is small-pox communicated to cows; or, finally, if cow-pox is natural to the cow.

*Experiment* 1. In January 1832 M. Fiard inoculated four cows from the matter obtained from the legs of a diseased horse; no pustules were produced.

*Experiment* 2. In January 1833 he inoculated seven cows on their teats with small-pox matter from a confluent case, without even producing inflammation of the punctures.

* This criticism is amply justified by a notice, which appeared since it was written, in a French journal, (*Gazette Méd.* 2 Avril, 1836,) acquainting us with the fact that the real cowpox had been discovered at Passy.—REV.

*Experiment* 3. In September 1833 he inoculated seven other cows with confluent small-pox matter without effect.

About this period the following experiments were made at the veterinary school at Alfort, to determine the value of the statements of Dr. Sunderland. Their object was to ascertain if small-pox could be communicated to cows by exposing them to the contact of the body and bed linen of those who had suffered from the disease. After having ascertained that there were no pustules of cow-pox on three cows, two of the animals were covered by a sheet and counterpane, and a third with a sheet, which had been used by small-pox patients at the Hôtel Dieu, and on which were observed stains made by the dried virus. These were fixed by belts to their bodies and remained on ten days and ten nights. During this time and twenty days afterwards the cows were seen every morning, but no trace of the disease was detected. Other pieces of linen were applied for seventeen days, and the teats rubbed with a shirt impregnated with virus, but no symptoms appeared though the cows were watched for the next month. In order to ascertain if the linen actually possessed contagious properties, the bodies of a dog and of a pig were each surrounded with one of the impregnated napkins which had not been used in the previous experiment; as each of these animals are known to contract the disease. The dog released himself from the napkin in twenty-four hours; the pig in ten minutes; and it was torn in pieces by other pigs with him. The two animals were examined every day for a month, and nothing appeared. Twenty-three days after the experiment a pig was observed to have a crop of pustules, like the natural small-pox in these animals, on the skin of his testicles, belly, and thighs; and eight days afterwards other pigs had a similar eruption, which ran through its regular stages. From these experiments at Alfort it appears, 1. That the small-pox could not be communicated to three cows by simple contact. 2. That it is probable the three pigs which destroyed with their teeth the napkin stained with virus, contracted the small-pox from the human subject. At the same time similar experiments were made at Rambouillet by Dr. Brunelle on cows and on four sheep: these are detailed, but the plan adopted was much the same as at Alfort, though more care was taken in procuring fresh small-pox virus. The animals contracted no disease.

Two individuals died of small-pox in a sort of cellar badly aired, and communicating with another cellar in which two cows were kept which were obliged to pass through the place where the sick persons lay. Although the clothes, &c. of the deceased persons remained more than fifteen days in the cows' stable, the animals were unaffected. A man lay ill of the small-pox in a stable with three cows who were not affected. Two setons steeped in the pus of small-pox were inserted into a young cow without inoculating her with the disease.

[Notwithstanding these results, the Academy does not regard the question as settled, but recommends it to further attention. The method by which cow-pox could be reproduced at will, would probably open a new field for research: and complete the benefits of Jenner's discovery.]

*La Lancette Française.* 2 *and* 5 *Mai*, 1835.

---

*On Carburet of Sulphur in Rheumatism.* By Professor OTTO, of Copenhagen.

CASE 1. A young man had been affected for six months with obstinate rheumatism of the lower limbs, the swelling of the knees and feet preventing all motion. The general health was not disturbed. The usual remedies, together with vapour-baths, had failed, when he consulted Dr. Otto, who directed him to continue the baths, and prescribed two drachms of carburet of sulphur in half an ounce of rectified spirit of wine, four drops of which to be taken every two hours; the lower limbs to be rubbed with a liniment consisting of two drachms of carburet of sulphur and half an ounce of olive-oil. The amendment was remarkably rapid: in less than four days the pain had so much diminished that he could rest his feet upon the ground, and five weeks afterwards the swelling, pain, and all the morbid symptoms had disappeared.

CASE 2. A sea captain, æt. 40, was attacked, after exposure to cold, with acute sciatica of the left thigh, preventing him from walking. The usual remedies were employed for a fortnight without success, when he saw Dr. Otto. The carburet of sulphur, internally and externally, was of immediate benefit. The pain diminished considerably in less than two days, and in eight days the patient was quite well.

CASE 3. A brazier, æt. 34, was attacked with acute rheumatic fever and sciatica of the left thigh, preventing motion. After the febrile symptoms ceased, the sciatica became chronic, which Dr. Otto treated with blisters, frictions, vinum antim., and tinct. guaiaci, but without benefit. On the fifth day he prescribed the carburet of sulphur internally and externally. In two days the pain diminished considerably; in four days he could move the limb with ease and walk tolerably; in ten days he was well.

CASE 4. This was an obstinate case of chronic rheumatism, in which the carburet was employed in connexion with calomel, guaiacum, &c., so that its effects could not be so distinctly traced. *Bibliothek for Læger*, 1835.

